# HOW CAREGIVERS USE BEST PROGRAMS FOR CAREGIVING TO FIND INTERVENTIONS IN THEIR COMMUNITIES

**DOI:** 10.1093/geroni/igae098.0033

**Published:** 2024-12-31

**Authors:** Rachel Cannon, David Bass, Morgan Minyo, Sara Powers, Zoe Fete, Megan Huth

**Affiliations:** Benjamin Rose, Cleveland, Ohio, United States; Benjamin Rose, Cleveland, Ohio, United States; Benjamin Rose Institute on Aging, Cleveland, Ohio, United States; Benjamin Rose, Cleveland, Ohio, United States; Benjamin Rose, Cleveland, Ohio, United States; Benjamin Rose, Cleveland, Ohio, United States

## Abstract

A new public version of Best Programs for Caregiving (BPC) launched in 2024 to help family and friend caregivers find evidence-based, dementia caregiving support programs that are available to them in their communities. The new version of BPC profiles healthcare and community organizations that are verified delivery sites of the 47 programs that are included in the professional version that launched in 2020. More than 200 organizations were verified to be delivering these programs and completed a survey requesting information about their offering of the program. The new version of BPC displays organization’s information on how caregivers can enroll in the program, types of help the program provides, program costs and eligibility requirements, how the program is delivered, and whether the program has been adapted for diverse groups. Caregivers can access Best Programs for Caregiving at bpc.caregiver.org and use their zip code to search for programs available to them both in-person and online. This presentation provides an overview of the interventions included in Best Programs for Caregiving and the data collection process, including a demonstration of how to use the site to find and compare evidence-based dementia caregiving programs.

